# Real-time ultrasound-guided catheterisation of the internal jugular vein: a prospective comparison with the landmark technique in critical care patients

**DOI:** 10.1186/cc5101

**Published:** 2006-11-17

**Authors:** Dimitrios Karakitsos, Nicolaos Labropoulos, Eric De Groot, Alexandros P Patrianakos, Gregorios Kouraklis, John Poularas, George Samonis, Dimosthenis A Tsoutsos, Manousos M Konstadoulakis, Andreas Karabinis

**Affiliations:** 1 Department of Intensive Care, General State Hospital of Athens, 154 Mesogeion Avenue, 11527 Athens, Greece; 2 Division of Vascular Surgery, University of Medicine and Dentistry of New Jersey, The University Hospital-150 Bergen Street Newark, NJ 07103 USA; 3 Academic Medical Center, Department of Vascular Medicine, University of Amsterdam Tafelbergweg 51 .1105 BD Amsterdam, The Netherlands; 4 Department of Cardiology, University Hospital of Heraklion, PO Box 1352 Stavrakia, Heraklion, Crete, Greece; 5 2nd Department of Propedeutic Surgery, University of Athens School of Medicine, Laiko General Hospital, 17 Agiou Thoma street-11527 Athens, Greece; 6 Department of Internal Medicine and Infectious Diseases, University of Crete, P. O. Box 2203, 71003 Heraklion, Greece; 7 'J. Ioannovic' Burn Center, General State Hospital of Athens, 154 Mesogeion Avenue, 11527 Athens, Greece; 8 1st Department of Propedeutic Surgery, University of Athens School of Medicine, Hipokrateion University Hospital,114 Vasilis Sofias Avenue 11527 Athens, Greece

## Abstract

**Introduction:**

Central venous cannulation is crucial in the management of the critical care patient. This study was designed to evaluate whether real-time ultrasound-guided cannulation of the internal jugular vein is superior to the standard landmark method.

**Methods:**

In this randomised study, 450 critical care patients who underwent real-time ultrasound-guided cannulation of the internal jugular vein were prospectively compared with 450 critical care patients in whom the landmark technique was used. Randomisation was performed by means of a computer-generated random-numbers table, and patients were stratified with regard to age, gender, and body mass index.

**Results:**

There were no significant differences in gender, age, body mass index, or side of cannulation (left or right) or in the presence of risk factors for difficult venous cannulation such as prior catheterisation, limited sites for access attempts, previous difficulties during catheterisation, previous mechanical complication, known vascular abnormality, untreated coagulopathy, skeletal deformity, and cannulation during cardiac arrest between the two groups of patients. Furthermore, the physicians who performed the procedures had comparable experience in the placement of central venous catheters (*p *= non-significant). Cannulation of the internal jugular vein was achieved in all patients by using ultrasound and in 425 of the patients (94.4%) by using the landmark technique (*p *< 0.001). Average access time (skin to vein) and number of attempts were significantly reduced in the ultrasound group of patients compared with the landmark group (*p *< 0.001). In the landmark group, puncture of the carotid artery occurred in 10.6% of patients, haematoma in 8.4%, haemothorax in 1.7%, pneumothorax in 2.4%, and central venous catheter-associated blood stream infection in 16%, which were all significantly increased compared with the ultrasound group (*p *< 0.001).

**Conclusion:**

The present data suggest that ultrasound-guided catheterisation of the internal jugular vein in critical care patients is superior to the landmark technique and therefore should be the method of choice in these patients.

## Introduction

Catheterisation of the internal jugular vein (IJV) is commonly attempted to obtain central venous access for haemodynamic monitoring, long-term administration of fluids, antibiotics, total parenteral nutrition, and haemodialysis in critical care patients. The safe puncture of the IJV is achieved by using anatomical landmarks on the skin's surface and thus passing the needle along the anticipated line of the vein. Many anatomic landmark-guided techniques for IJV puncture have been described since 1966 [1-4]. Complications, including death, are influenced by patient factors such as body mass index (BMI), site of attempted access, and operator experience [5-7]. Furthermore, inability to cannulate the IJV may occur in up to 19.4% of cases [6].

It has been suggested that ultrasound guidance could be beneficial in placing central venous catheters (CVCs) by improving the success rate, reducing the number of needle passes, and decreasing complications [8-12]. Also, employment of ultrasound imaging may identify patients in whom central venous access may be more difficult and/or in whom consequences of complications could be more serious [13]. Although the ultrasound method has compared favourably with the landmark technique, its widespread use has been hampered by the impracticality of specially designed ultrasound devices or sterile scanner manipulation, unavailability of equipment, and lack of trained personnel. Furthermore, previous studies of ultrasound location of vessels followed by subsequent catheter placement with landmark techniques found no advantages over standard landmark techniques [7]. However, few prospective studies exist comparing the technique itself of ultrasound-guided central venous cannulation versus the landmark method in critical care patients [14]. This prospective study was designed to compare the real-time ultrasound-guided approach with the landmark technique in the cannulation of the IJV in critical care patients.

## Materials and methods

### Patients

This prospective study was conducted from January 2000 to December 2006 in 900 mechanically ventilated critical care patients (the average number of patients hospitalised per year in our unit is 170). The patients were randomly assigned on a one-to-one ratio. Randomisation was performed by means of a computer-generated random-numbers table, and patients were stratified with regard to age, gender, and BMI. Block randomisation was used to ensure equal numbers of patients in the above groups [15]. All physicians and other research personnel were blinded to the randomisation schedule and the block size. Family members provided written, informed consent for all patients. The study was conducted in accordance with the principles outlined in the Declaration of Helsinki and was approved by the Institutional Ethics Committee.

Successful placement of the CVC was assessed by a chest x-ray obtained after the procedure. Mechanical complications were defined as carotid artery puncture, skin haematoma, pneumothorax, haemothorax, and catheter malposition. Carotid artery puncture was noted by forceful pulsatile expulsion of bright red blood from the needle. All mechanical complications were evaluated clinically, by a chest x-ray, and by means of ultrasonography where appropriate. In most patients in whom the first attempt (one pass of the introducing needle) at catheterisation failed, another physician performed the next attempt. If a catheter was misplaced, the position was corrected either by a 'power flash' (a rapid infusion of 10 ml of saline solution pushed through the catheter with a syringe) or by manipulation of the catheter under fluoroscopic guidance. Pneumothorax was treated with tube thoracostomy if it was symptomatic or progressive or if more than 20 percent of the interface between the lung and the chest wall was separated.

### Methods

#### Landmark technique

For the landmark technique, the patient was placed in a supine position. The skin at the top of the triangle between the sternal and clavicular head of the sternocleidomastoid muscle was degreased with acetone and prepared in a sterile fashion with povidone-iodine. Then, the area was anaesthetised with a 1% xylocaine solution with a 22-gauge needle. Physicians were encouraged to locate the IJV with this 'finder' needle connected to a 2-ml syringe as the needle was advanced through the skin at a 45° angle in the direction of the right or the left nipple (for cannulation of the right or the left IJV, respectively). The return of venous blood into the syringe attached to the needle confirmed entry into the vessel, and the finder needle was used to guide a 19-gauge, 10-cm needle connected to a 10-ml syringe (Arrow Howes; Arrow International, Inc., Reading, PA, USA) [16]. A guidewire was then placed through the needle into the vein, and the needle was removed. A catheter or sheath was placed over the wire and advanced into the IJV.

#### Real-time ultrasound-guided method

**Figure 1:**
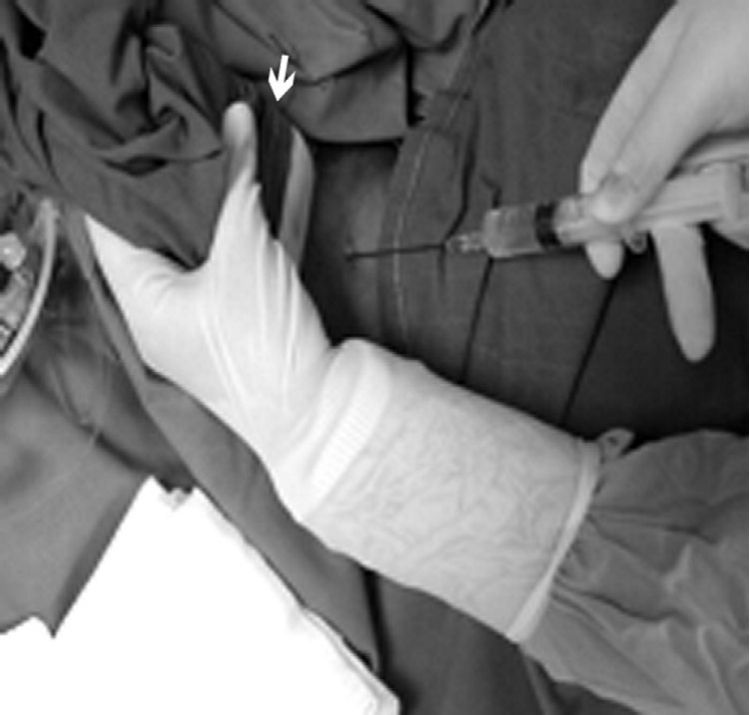
The transducer is placed over the groove parallel and superior to the right clavicle (arrow).

**Figure 2:**
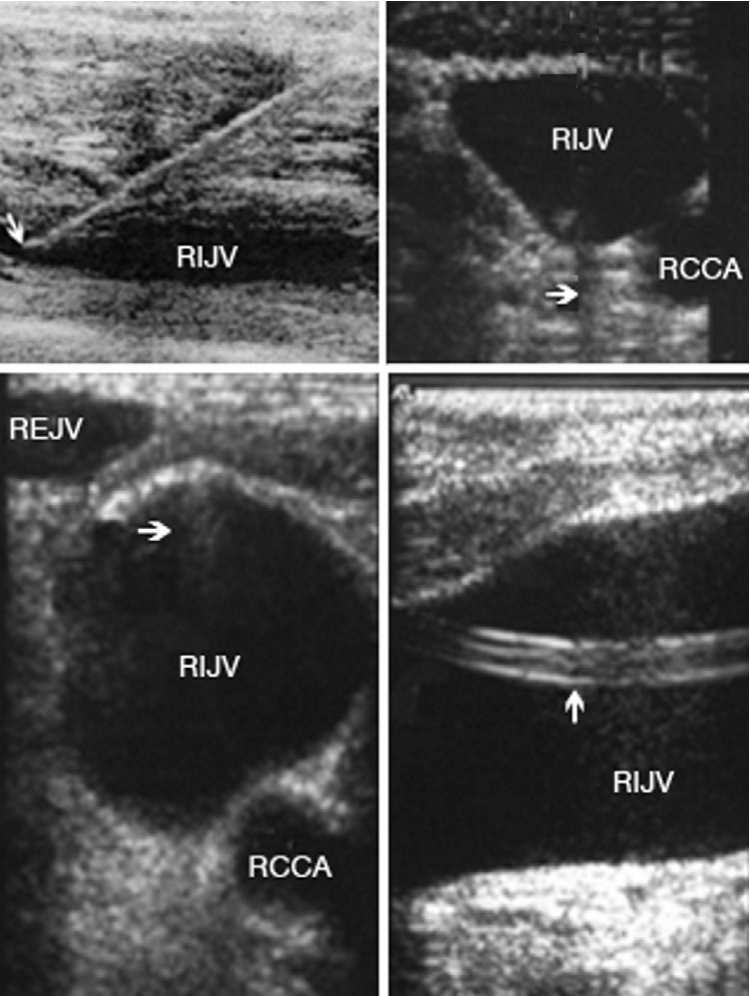
**(Top left): **Visualisation of the needle entering the anterior wall of the right internal jugular vein (RIJV) (longitudinal axis) (arrow). **(Bottom left): **Visualisation of the guidewire entering the venous lumen (arrow). **(Top right): **Visualisation of the needle entering the venous lumen (transverse axis). The black line behind the needle is the echo shadow (arrow). **(Bottom right): **Sagittal view of the neck, showing the catheter placed within the lumen (arrow). RCCA, right common carotid artery; REJV, right external jugular vein.

The neck area was prepared and draped sterilely with the patient supine as described above. A 7.5-MHz linear-array ultrasound probe connected to a real-time ultrasound unit (ATL 3500; Philips Medical Systems, Andover, MA, USA), and focused at 6.5-cm depth, was covered with ultrasonic gel and wrapped in a sterile plastic sheath. By wrapping the transducer in a sterile sheath, its use in consecutive patients is facilitated (Figure 1). Standard ultrasound two-dimensional (2D) imaging was used to measure the depth and calibre of the IJV, evaluate its patency and compressibility, and identify whether there were any thrombi in the vein. In cases of pre-existing thrombus formation and/or failure to gain access due to trauma or other anatomical anomalies, the IJV on the contralateral side was catheterised. Catheterisation was performed under continuous dynamic observation of real-time 2D images obtained by placing the transducer parallel and superior to the clavicle, over the groove between the sternal and clavicular heads of the sternocleidomastoid muscle. This readily visualised the IJV, the external jugular vein, and the carotid artery (Figure 2). A 19-gauge, 10-cm needle (Arrow Howes; Arrow International, Inc.) was advanced through the skin under ultrasound guidance into the IJV. A guidewire was then placed through the needle into the vein, and the needle was removed. A catheter or sheath was placed over the wire and advanced into the IJV (Figure 2). The needles and guidewires used were all standard components of catheterisation kits and were not modified versions for use with ultrasound. All ultrasound-guided and landmark-guided catheterisations were performed by well-trained attending cardiologists, intensivists, and surgeons with similar experience (10 years of experience in IJV catheter placements, *p *= non-significant) to minimise the effect of operator experience on the success rate and the rate of mechanical complications. Furthermore, the physicians who performed the ultrasound-guided method were well trained and had at least 5 years of experience in performing this method.

### Data collection and statistical analysis

Forms containing patients' characteristics and all the pertinent fields for each technique were filled out in a timely fashion, and data were entered in a customised database. The following data were also recorded: side of catheterisation (either left or right) and the presence of risk factors for difficult venous cannulation such as prior catheterisation, limited sites for access attempts (other catheters, pacemaker, and local surgery or infection), previous difficulties during catheterisation (more than three punctures at one site, two sites attempted, and failure to gain access), previous mechanical complication, known vascular abnormality, untreated coagulopathy (international normalisation ratio >2, activated partial thromboplastin time >1.5, and platelets <50 × 10^9 ^per litre), skeletal deformity, and cannulation during cardiac arrest [5,13]. The outcomes assessed were the access time, the average number of attempts before successful placement (defined as separate skin punctures), the success of placement, the rate of mechanical complications, and the incidence of CVC-associated blood stream infection (CVC-BSI). Access time was defined as the time between penetration of skin and aspiration of venous blood into the syringe. When a multiple pass was performed, only the time from skin contact of the first needle to IJV cannulation was taken into account. This was made to ensure an objective comparison between the two methods. Counting the entire procedural time would have clouded the issue because other parameters such as nursing performance could affect the measurement. Preparation times for both techniques were quite similar. The access time was measured in seconds by stopwatch by other physicians, and the number of attempts and complications were recorded. It is of note that every effort was made to ensure the application of evidence-based catheter insertion practices in both methods [17]. All patients were receiving antibiotic treatment during the study period. CVC-BSIs were defined as only those blood stream infections for which other sources were excluded by careful examination of the patient record and in which a culture of the catheter tip demonstrated substantial colonies of an organism identical to those found in the bloodstream [17].

Data were expressed as mean ± standard deviation. The Student *t *test for independent means, χ^2 ^analysis, or Fisher exact test where appropriate were used to identify differences between the two groups. Correlations between continuous variables were assessed using the Pearson correlation coefficient. For ordinal data, the Spearman rank correlation was used. A *p *value (two-sided in all tests) of <0.05 was considered significant. SPSS software, version 11.0, was used (SPSS Inc., Chicago, IL, USA).

## Results

Baseline characteristics of the study population are presented in Table 1. There were no significant differences between the two groups of patients in gender ratio, age, BMI, or side of catheterisation or in the presence of risk factors for difficult venous cannulation such as prior catheterisation, limited sites for access attempts, previous difficulties during catheterisation, previous mechanical complication, known vascular abnormality, untreated coagulopathy, skeletal deformity, and cannulation during cardiac arrest (Table 1).

**Figure 3:**
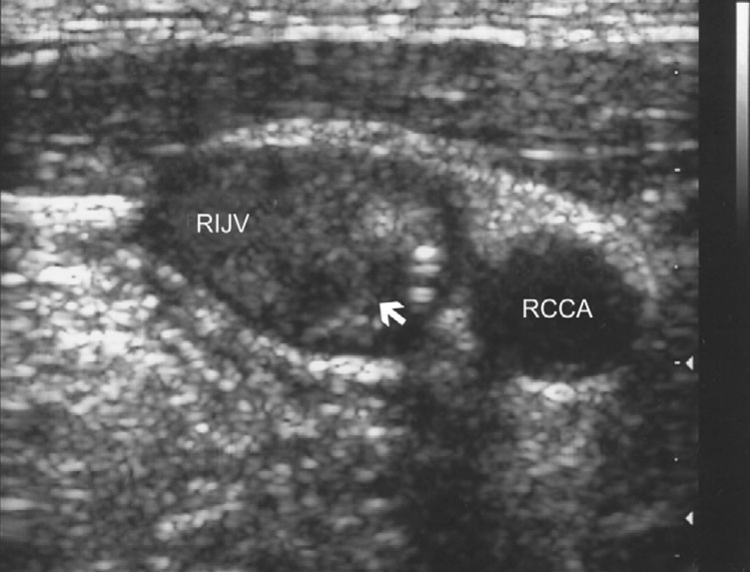
Thrombus visualised within the right internal jugular vein (RIJV) (arrow). The vessel could not be compressed. RCCA, right common carotid artery.

In all except 34 patients in the ultrasound group, the IJV was visualised and cannulated. In these 34 patients who had had prior surgery and/or prior cannulations, ultrasound imaging clearly detected the presence of thrombus (Figure 3); thus, during the same session, the IJV on the contralateral side of the neck was catheterised instead. Furthermore, 25 patients in the landmark group in whom catheterisation was unsuccessful were converted to the ultrasound method. Thrombosis was identified in 20 cases (which led to formal anticoagulation of these patients) and anatomical variation of the IJV in five patients, and these were very likely the reasons for which the landmark method failed. During the ultrasound-guided procedure, the IJV can be compressed completely by the needle before the vessel is actually penetrated. Then, the needle must be advanced a little deeper and retracted slightly to be positioned in the center of the lumen. In accordance with this, we have used 2D ultrasound images recorded on both transverse and longitudinal axes during the same session (Figure 2). The 2D image provides important information about venous location and size. Visualisation of the IJV on the transverse axis was particularly useful for catheterisation, especially when the vein diameter was small, whereas visualisation of the vein on the longitudinal axis provided a clear image of both walls of the vessel (the actual vein puncture using either the longitudinal or the transverse axis of the 2D image was left to the discretion of the operator). Also, using this approach, a single-wall puncture can be made by observing the point at which the needle first indents the anterior wall of the IJV. A short stabbing motion of the needle at this point will tend to puncture the anterior wall without opposing it to the posterior wall, thereby avoiding a double-wall puncture (Figure 2). Single-wall punctures were achieved in all cases using ultrasound guidance.

Interestingly, five significant anatomical variants between the IJV and common carotid were observed in the ultrasound group. In 188 (41.7%) cases, the IJV was anterior and lateral to the artery; in 120 (26.6%) cases, it was laterally located; and in 72 (16%) cases, it was directly anterior to the common carotid artery. In the remaining cases, the IJV was anterior and medial to the common carotid artery in 53 (12.6%) cases and directly medial to the artery in 17 (3.7%) cases.

Results using the landmark technique are in sharp contrast to those obtained by the ultrasound method and are presented in Table 2. Average access time and number of attempts were both significantly reduced using ultrasound compared with the landmark technique (*p *< 0.001) (Table 2). The success rate was significantly lower and the rate of mechanical complications was significantly higher in the landmark group of patients as compared with the ultrasound group (*p *< 0.001) (Table 2). Furthermore, in the landmark group, four cases of haemothorax and four cases of pneumothorax which required therapeutic intervention occurred, but no such complication was observed in the ultrasound group. Interestingly, the present data showed a significantly increased number of CVC-BSIs in the landmark group compared with those documented in the ultrasound group (*p *< 0.001) (Table 2). It is of note that the number of CVC-BSIs was positively correlated to the number of needle passes in the total study population (*r *= 0.65, *p *< 0.001). The type of microorganisms responsible for the CVC-BSIs in the ultrasound group of patients versus those responsible for the CVC-BSIs in the landmark group was similar: coagulase-negative *Staphylococci *(48.6% versus 56.8%, respectively), *Staphylococcus aureus *(27.02% versus 24.1%), *Enterococus *species (13.5% versus 10.3%), *Escherichia coli *(2.7% versus 3.4%), *Enterobacter *(2.7% versus 1.7%), *Pseudomonas aeruginosa *(2.7% versus 1.7%), and *Candida *species (2.7% versus 1.7%). However, the incidence of coagulase-negative *Staphylococci *was significantly higher in the landmark group of patients compared with the ultrasound group (*p *< 0.05). Finally, the incidence of co-morbidities, including cancer, that might have affected the patients' immune status was similar in the ultrasound group as compared with the landmark group (10% versus 11%, *p *= non-significant, respectively).

## Discussion

The use of CVCs may be associated with adverse effects that are both hazardous to patients and expensive to treat [18]. Mechanical complications are reported to occur in 5% to 19% of patients, infectious complications in 5% to 26%, and thrombotic complications in 2% to 26% [19,20]. These complications increase in association with several characteristics, including patient anatomy (for example, morbid obesity, cachexia, or local scarring from surgery or radiation treatment), patient setting (for example, patients receiving mechanical ventilation or during emergencies such as cardiac arrest), co-morbidities, and operator's experience [6-8,13]. Real-time ultrasound guidance of CVC insertion provides the operator with visualisation of the desired vein and the surrounding anatomic structures prior to and during the insertion of the catheter. This method appears to improve the success rate and decrease the complication rate associated with CVC placement [8,11,12]. The present data further support the superiority of real-time ultrasound-guided IJV cannulation as compared with the landmark technique in mechanically ventilated, critical care patients.

Using the landmark method, we showed a successful IJV cannulation rate of 94.4%, which is in accordance with success rates documented in previous reports ranging from 85% to 99% [2,3,6,12,21,22]. The incidence of carotid puncture using the landmark method (10.6%) was comparable with larger studies [12,21] but higher than those reported (3% to 6%) in smaller series [2,6]. Also, the incidence of haematoma and pneumothorax (8.4% and 2.4%, respectively) using the landmark method was in the range of previous studies [2,6,12,21].

The incidence of mechanical complications using the ultrasound-guided technique was negligible, which is in agreement with previous reports [12,14,22]. Using ultrasound guidance, the incidence of carotid puncture and haematoma was very low, and (as shown before) no cases of haemothorax and/or pneumothorax were observed [12,22]. Interestingly, in several patients in whom carotid puncture occurred, it was noted that the IJV was overlying the carotid artery rather than being more lateral. To avoid the carotid artery in these cases, a sideway approach of puncturing the IJV was used instead of the perpendicular approach.

It is of note that the most favourable outcomes associated with real-time ultrasound guidance as compared with the landmark technique were found in studies of inexperienced observers [22-24]. We showed the superiority of the ultrasound method in a study in which all observers had comparable experience in CVC placement. The patients who underwent the ultrasound-guided cannulation and those who underwent the landmark-guided cannulation of the IJV were comparable in everything that is pertinent to this procedure, including the presence of risk factors for difficult venous cannulation. To the best of our knowledge, this is the first time that such a controlled comparison has been done between the two methods of IJV cannulation. This is important because no other factors should have affected the results and therefore the only determining factor was the technique itself.

The clinical notion that the additional equipment and manipulation associated with the ultrasound method might have increased the rate of catheter-related infection was not confirmed by the present data. We found that the incidence of CVC-BSI in the ultrasound group of patients was significantly lower compared with that documented in the landmark group. The number of CVC-BSIs was significantly correlated to the number of needle passes in the total study population. We could speculate that repeated attempts might lead to a breakdown of aseptic technique and more colonisation of skin-related pathogens [17]. The above findings may be of clinical importance for two reasons. First, it is well documented that CVC-BSI is a common problem in the management of the critical care patient [17]. Second, catheters inserted into the IJV have been associated with higher risk for infection than those inserted into the subclavian or femoral vein [25,26]. In this study, the incidence of bacterial strains implicated in CVC-BSI was similar in patients of the landmark group as compared with patients of the ultrasound group, except for a significantly higher incidence of coagulase-negative *Staphylococci *which was documented in the landmark group. A possible explanation for the above observation may be the increased access time and number of average attempts which were documented in the landmark group compared with the ultrasound group. Furthermore, the density of skin flora at the catheter insertion site is a major risk factor for CVC-BSI [25]. Some authors recommend that, to reduce the risk for infection, CVCs be placed in a subclavian site instead of a jugular or femoral site [27,28]. However, no randomised trial has satisfactorily compared infection rates for catheters placed in jugular, subclavian, and femoral sites [17,19].

Thrombosis was detected in 54 patients, 20 of whom were in the landmark group and were converted to the ultrasound group. Hence, ultrasound imaging is an important tool in identifying cases of pre-existing thrombus formation and anatomic variations in the IJV location, thus facilitating safer and more successful catheterisation of the vessel. In a previous study, ultrasonography imaging detected venous thrombosis in 33% of critical care patients; in approximately 15% of these patients, the thrombosis was catheter-related [29]. Also, attempts to cannulate thrombotic veins usually are unsuccessful even when the anatomy is normal [13]. It was suggested that the risk of catheter-related thrombosis varies according to the site of insertion. In one report, catheter-related thrombosis occurred in 21.5% of the patients with femoral venous catheters and in 1.9% of those with subclavian venous catheters [19]. In an observational study, the risk of thrombosis associated with internal jugular insertion was approximately four times the risk associated with subclavian insertion [30].

We used only standard components of catheterisation kits and not modified versions for use with ultrasound. Other groups have used more sophisticated, and thus more expensive, ultrasound-guided cannulation devices [31]. The major impediments to the widespread implementation of the above method are the purchase costs of the ultrasound machines. However, past studies have provided sufficient economic arguments supporting the notion that ultrasound-guided central venous cannulation is cost-effective [31,32].

### Technical considerations and study limitations

The ultrasound method is technically demanding, requiring well-trained operators and adequate experience in performing it [18]. The benefits of this method may not accrue until after an initial learning period for operators already experienced in the landmark technique [31]. We employed the visualisation of the IJV and of neighboring anatomical structures on both the longitudinal and transverse axes during real-rime ultrasound-guided cannulation. This approach offers the advantage of better positioning of the needle, clear visualisation of the procedure, avoidance of double-wall puncture, and precise placement of the catheter in the vessel lumen [33].

### Perspectives and conclusions

In addition to real-time ultrasound guidance, other approaches may reduce the risks associated with CVC insertion. Peripheral venous cannulation under ultrasound may be an acceptable substitute for CVC placement for certain indications (long-term i.v. access or parenteral nutrition) [34,35]. Alternative methods for teaching CVC insertion may employ computerised technologies for simulations. Haptic techniques use virtual reality models to create immersive simulated environments that recreate the sensation of performing a procedure [36]. However, we believe that ultrasound imaging is a readily available technology and may be employed by inexperienced operators to facilitate the placement of a CVC and by experienced operators to improve the safety of the procedure.

## Conclusion

This study showed that real-time ultrasound-guided catheterisation of the IJV offers the advantage of a shorter access time and a reduced number of successful attempts compared with the landmark-guided technique. Also, this method has a lower mechanical complication rate and may result in a lower incidence of CVC-BSI compared with the landmark technique. There is no doubt that critically ill patients benefit most from the above advantages of the ultrasound method [8,11,14,18,19,30].

## Key messages

• The visualisation of the IJV and of neighboring anatomical structures on both the longitudinal and transverse axes ensures the avoidance of a double-wall puncture during catheterisation.

• Ultrasound-guided cannulation of the IJV offers the advantage of a shorter access time and a reduced number of successful attempts.

• Ultrasound-guided cannulation of the IJV has a lower mechanical complication rate and may result in a lower incidence of CVC-BSI compared with the landmark technique.

## Abbreviations

BMI = body mass index; CVC = central venous catheter; CVC-BSI = central venous catheter-associated blood stream infection; IJV = internal jugular vein; 2D = two-dimensional.

## Competing interests

The authors declare that they have no competing interests.

## Authors' contributions

DK conceived of this study, participated in the design of the study, performed both methods in the intensive care unit setting, and drafted the manuscript. NL participated in the design of the study, provided expert advice concerning both methods, and performed the statistical analysis. EDG participated in the design of the study, provided expert advice concerning the ultrasound method, and helped to draft the manuscript. APP participated in the design of the study, carried out both methods in the intensive care unit setting, and helped to draft the manuscript. GK performed both methods in the intensive care unit setting and helped to draft the manuscript. JP performed both methods in the intensive care unit setting and helped to draft the manuscript. GS participated in the design of this study and provided expert advice concerning catheter associated blood stream infections. DAT participated in the design of this study and performed both methods in the intensive care unit setting. MMK participated in the design of the study, performed both methods in the intensive care unit setting, and helped in the statistical analysis. AK participated in the design of the study and coordination and helped to draft the manuscript. All authors read and approved the final manuscript.

## Figures and Tables

**Table 1:** Characteristics of the total study population

Characteristics	Ultrasound group (*n *= 450)	Landmark group (*n *= 450)
Age (years)^a^	58.3 ± 10.3	59 ± 9.5
Gender (male/female ratio)^a^	0.56 ± 0.4	0.6 ± 0.4
Side of catheterisation (left/right)	222/228	218/232
Body mass index (kg/m^2^)^a^	24.1 ± 5.3	23.7 ± 5.9
Prior catheterisation	85 (18.8%)	76 (16.8%)
Limited sites for access attempts	51 (11.3%)	55 (12.2%)
Previous difficulties during catheterisation	44 (9.7%)	40 (8.8%)
Previous mechanical complication	18 (4%)	20 (4.4%)
Known vascular abnormality	4 (0.8%)	3 (0.6%)
Untreated coagulopathy	25 (5.5%)	24 (5.3%)
Skeletal deformity	15 (3.3%)	13 (2.8%)
Cannulation during cardiac arrest	31 (6.8%)	35 (7.7%)

^a^Values are presented as mean ± standard deviation.

**Table 2:** Outcome measures in the ultrasound group versus the landmark group of patients

Outcome measures	Ultrasound group (*n *= 450)	Landmark group (*n *= 450)
Access time (seconds)	17.1 ± 16.5 (11.5 to 41.4)^a^	44 ± 95.4 (33.2 to 77.5)
Success rate	450 (100%)^a^	425 (94.4%)
Carotid puncture	5 (1.1%)^a^	48 (10.6%)
Haematoma	2 (0.4%)^a^	38 (8.4%)
Haemothorax	0 (0%)^a^	8 (1.7%)
Pneumothorax	0 (0%)^a^	11 (2.4%)
Average number of attempts	1.1 ± 0.6 (1.1 to 1.9)^a^	2.6 ± 2.9 (1.5 to 6.3)
CVC-BSI	47 (10.4%)^a^	72 (16%)

^a^Comparison of the outcome measures between the ultrasound group and the landmark group of patients (*p *< 0.001). Access time and average number of attempts are expressed as mean ± standard deviation (95% confidence interval). Success rate, carotid puncture, haematoma, haemothorax, pneumothorax, and CVC-BSI are expressed as the absolute number of patients and percentage of their group. CVC-BSI, central venous catheter-associated blood stream infection.
